# Rapid‐Onset Amoxicillin–Clavulanate Liver Injury Requires Stronger Exclusion of Alternative Causes and Fuller Causality Reporting in Atypical DILI Case Reports

**DOI:** 10.1002/ccr3.73369

**Published:** 2026-08-28

**Authors:** Muhammad Abdullah Awan, Hammad‐ud‐din Raza

**Affiliations:** ^1^ Wah Medical College, National University of Medical Sciences Wah Cantonment Pakistan

**Keywords:** amoxicillin‐clavulanate, causality assessment, cholestatic hepatitis, drug‐induced liver injury, infectious mononucleosis, RUCAM

## Abstract

Rapid‐onset drug‐induced liver injury after amoxicillin–clavulanate is clinically important, but atypical latency requires particularly careful exclusion of alternative causes. Future reports should provide complete exposure chronology, infectious and autoimmune exclusion, concomitant‐drug assessment, RUCAM itemization, and longer biochemical follow‐up before assigning strong causality to amoxicillin–clavulanate.

Dear Editor,

We read with great interest the case report by Khadka et al., titled “Rapid‐Onset Drug‐Induced Liver Injury Following Amoxicillin–Clavulanate: A Case Report,” published in Clinical Case Reports [1]. The authors describe a 32‐year‐old female who developed jaundice and deranged liver tests after only two doses of amoxicillin–clavulanate prescribed for acute tonsillopharyngitis [1]. The report is valuable because it reminds clinicians that drug‐induced liver injury (DILI) should remain in the differential diagnosis even in resource‐limited settings and after apparently brief antibiotic exposure.

However, one interpretive point deserves further emphasis: when the latency is unusually short, the exclusion of alternative causes and the strength of causality reporting become even more important. In the reported case, the patient initially had exudative tonsillopharyngitis, fever, fatigue, monocytosis, thrombocytopenia, and jaundice [1]. These features overlap with viral illnesses that may produce hepatitis or cholestatic biochemical changes. The authors acknowledge that Epstein–Barr virus, cytomegalovirus, and autoimmune hepatitis testing could not be performed because of resource limitations [1]. This limitation does not invalidate the case, but it should temper the certainty with which amoxicillin–clavulanate is framed as the cause.

A second issue is concomitant medication. The patient received both amoxicillin–clavulanate and paracetamol, and both were stopped simultaneously when liver injury was suspected [1]. Although the time course may be more compatible with amoxicillin–clavulanate, future atypical DILI reports should clearly document dose timing, cumulative dose, any self‐medication before presentation, and the rationale for excluding each concomitant drug. This is especially relevant when improvement follows withdrawal of more than one medication.

The authors appropriately used RUCAM, but the score was + 5, categorizing the association as “possible” rather than probable or highly probable [1]. This is a useful and honest finding. Nevertheless, the language of future reports should remain aligned with the causality category. A possible RUCAM score supports clinical suspicion, but it also indicates residual uncertainty, particularly when EBV, CMV, and autoimmune markers are unavailable.

The report would also be strengthened by a more granular chronology. Because onset after two doses is the central novelty, future reports should specify the exact interval from first dose to symptom onset, from second dose to jaundice, and from drug withdrawal to each biochemical improvement. Similarly, longer follow‐up beyond the early fall in bilirubin and enzymes would help confirm sustained recovery and clarify whether the pattern remained cholestatic, mixed, or evolved over time [1].

Overall, Khadka et al. present an educational case that appropriately highlights the need for vigilance regarding amoxicillin–clavulanate‐associated DILI [1]. The broader lesson is that atypically rapid DILI should be reported with a stricter causality framework. In resource‐limited settings, where confirmatory tests may be unavailable, transparent reporting of what was excluded, what could not be excluded, and how causality was graded is essential for making case‐based evidence clinically reproducible.

This case usefully highlights possible rapid‐onset amoxicillin–clavulanate‐associated liver injury. However, future atypical DILI case reports should include clearer exposure chronology, fuller evaluation of infectious and autoimmune alternatives, explicit assessment of concomitant drugs, careful alignment of causal language with RUCAM scoring, and longer biochemical follow‐up. These details would strengthen diagnostic confidence while preserving the clinical value of early recognition and drug withdrawal.

## Author Contributions

**Muhammad Abdullah Awan:** conceptualization, data curation, formal analysis, project administration, writing – original draft, writing – review and editing. **Hammad‐ud‐din Raza:** formal analysis, writing – original draft, writing – review and editing, data curation.

## Funding

The authors have nothing to report.

## Ethics Statement

The authors have nothing to report.

## Consent

The authors have nothing to report.

## Conflicts of Interest

The authors declare no conflicts of interest.

## Data Availability

Data sharing not applicable to this article as no datasets were generated or analysed during the current study.
